# The symbiotic alga *Trebouxia* fuels a coherent soil ecosystem on the landscape scale in the Atacama Desert

**DOI:** 10.1186/s40793-024-00601-5

**Published:** 2024-08-09

**Authors:** Patrick Jung, Rebekah Brand, Laura Briegel-Williams, Lina Werner, Emily Jost, Guillaume Lentendu, David Singer, Rujuta Athavale, Dennis J. Nürnberg, Fernando D. Alfaro, Burkhard Büdel, Michael Lakatos

**Affiliations:** 1 Department of Integrative Biotechnology, University of Applied Sciences Kaiserslautern, Pirmasens, Germany; 2 Laboratory of Soil Biodiversity, Université de Neuchâtel, Neuchâtel, Switzerland; 3 Soil Science and Environment Group, Changins, HES-SO University of Applied Sciences and Arts Western Switzerland, Nyon, Switzerland; 4 Institute for Experimental Physics, Freie Universität Berlin, Berlin, Germany; 5 Dahlem Centre of Plant Sciences, Freie Universität Berlin, Berlin, Germany; 6 GEMA Center for Genomics, Ecology and Environment, Universidad Mayor, Santiago, Chile; 7 Biology, Rhineland-Palatinate Technical University Kaiserslautern Landau, Kaiserslautern, Germany

**Keywords:** Lichens, Mycobionts, Photobionts, Caliciaceae, Green algae

## Abstract

Biocrusts represent associations of lichens, green algae, cyanobacteria, fungi and other microorganisms, colonizing soils in varying proportions of principally arid biomes. The so-called grit crust represents a recently discovered type of biocrust situated in the Coastal Range of the Atacama Desert (Chile) made of microorganisms growing on and in granitoid pebbles, resulting in a checkerboard pattern visible to the naked eye on the landscape scale. This specific microbiome fulfills a broad range of ecosystem services, all probably driven by fog and dew-induced photosynthetic activity of mainly micro-lichens. To understand its biodiversity and impact, we applied a polyphasic approach on the phototrophic microbiome of this biocrust, combining isolation and characterization of the lichen photobionts, multi-gene phylogeny of the photobionts and mycobionts based on a direct sequencing and microphotography approach, metabarcoding and determination of chlorophyll_a+b_ contents. Metabarcoding showed that yet undescribed lichens within the Caliciaceae dominated the biocrust together with *Trebouxia* as the most abundant eukaryote in all plots. Together with high mean chlorophyll_a+b_ contents exceeding 410 mg m^−2^, this distinguished the symbiotic algae *Trebouxia* as the main driver of the grit crust ecosystem. The trebouxioid photobionts could be assigned to the I (*T. impressa*/*gelatinosa*) and A (*T. arboricola*) clades and represented several lineages containing five potential species candidates, which were identified based on the unique phylogenetic position, morphological features, and developmental cycles of the corresponding isolates. These results designate the grit crust as the only known coherent soil layer with significant landscape covering impact of at least 440 km^2^, predominantly ruled by a single symbiotic algal genus.

**Supplementary Information:**

The online version contains supplementary material available at 10.1186/s40793-024-00601-5.

## Introduction

One of the most abundant complex and natural microbial systems are biological soil crusts (biocrusts), which occur globally on 12% of the terrestrial surface, especially in water-limited environments where vascular plants are restricted [1]. They are considered pioneer communities in harsh environments such as polar- and non-polar deserts because they are composed of a predominantly photoautotrophic, poikilohydric group of organisms called cryptogams (meaning ‘hidden reproduction’): green algae, cyanobacteria, bryophytes as well as lichens together with heterotrophic bacteria and fungi [2]. All together, these organisms can colonize the first millimeters of soil and concatenate particles with their extracellular polymeric substances, filaments, and root-like structures, resulting in a physical, biotic crust [3]. Depending on soil characteristics, biome and also microbial community composition biocrusts can be divided into several types according to functional groups and/or the dominant organism [4–6]. Biocrusts from polar regions, for example, are often dominated by bryophytes [7–9], and those of the Negev and Sahara Desert by cyanobacteria [10, 11] while lichens occurring in small patches are the predominant biocrust organisms of the Colorado Plateau [12], or in the Namib Desert [13]. All of these biocrust types have significant and multifactorial influences on their environment by fulfilling several ecosystem services such as erosion prevention [14], but also modulating nitrogen-, phosphorus- and carbon cycles [15]. In addition, biocrusts exert strong influences on hydrological processes in drylands by modifying numerous soil properties that affect water retention and movement in soils such as infiltration, runoff, soil moisture and evaporation [16]. As ecosystem engineers, they can be pioneers in the colonization of inert substrates, where they play a crucial role in ecological succession by promoting initial soil formation or priming [17, 18], often resulting in the subsequent establishment of vascular plants [19, 20]. All of these important ecosystem services are the result of the extremotolerant character of cryptogams which allows them to withstand extreme temperature amplitudes and low precipitation through desiccation tolerance [21–23], but also their complex organismic [24, 25] and symbiotic nature spanning domains, kingdoms, and phyla [26, 27].

Among such symbiotic associations of biocrusts are the lichens. In general, they represent an iconic example of symbiosis where green algae and/or cyanobacteria (photobionts) team up with a fungus (mycobiont), and include other symbiotic microorganisms, which significantly contribute to the symbiosis (e.g., [28–30]). The distribution and habitat preferences of lichens are shaped by the requirements of all symbionts forming the holobiont [31], and according to this the symbionts allow their hosts to live in habitats from which they would be otherwise excluded. Their complexity has recently been acknowledged by redefining lichens as self-sustaining complex micro-ecosystems [32], albeit the role of other microorganisms—apart from the mycobiont and photobiont—are not demonstrably known [33, 34]. The debate about this terminology exemplifies how crucial the understanding of such mycobiont-photobiont relationships are, adding another diversity level to biocrusts. However, lichens and especially their photobionts associated with biocrusts have rarely been investigated [8, 35], which might be because such lichens often occur in isolated patches due to their mainly squamulose growth forms. This isolated appearance is the case for *Psora decipiens* from the Colorado Plateau [12] or *Teloschistes capensis* from the Namib Desert [13], while, for example, cyanobacteria fill the lichen-free spaces between them resulting in a much higher cohesive coverage than the single lichens. Consequently, a patchy presence of lichens within biocrusts also limits the appearance as well as the functional role of the lichen photobionts. Such lichen photobionts are for example members of the green algal genus *Trebouxia*, one of the most abundant lichen photobionts [36–38], which has rarely been found free living [39–42]. The taxonomic system for the diverse photobiont genus *Trebouxia* was recently updated, dividing the genus into four main lineages: A- (*T. arboricola*), C- (*T. corticola*), I- (*T. impressa* / *T. gelatinosa*) and S-clade (*T. simplex* / *T. jamesii*). This new framework is based on historical taxonomic work on isolated *Trebouxia*-species [43–47] in combination with modern multilocus phylogenies and morpho-anatomical features such as chloroplast architecture [48–50]. Following this approach, species-level lineages within each of the four major clades can also be designated, including potential species candidates, based on the phylogenetic position and morphology of representative isolates [51, 52].

So far, such a comprehensive approach has not been carried out for any biocrust environment and provides an interesting opportunity to evaluate the role of photobionts in a novel and unique lichen-dominated grit crust environment from the Atacama Desert [53]. This microbiome has been described as a transitional stage between a saxicolous community (growing on rocks) and a biocrust because the microorganisms are colonizing granitoid pebbles called grits of about 6 mm in diameter and not a fine substrate equivalent to soil [3, 53]. The microorganisms creating the grit crust reach comparably high densities, resulting in a blackish coloration of the grits, so that the biocrust’s extension appears as a checkerboard pattern on the landscape scale covering about 80% of the 440 km^2^ encompassing National Park Pan de Azucar [53]. There, abiotic conditions are not as harsh as in hyper-arid parts of the Atacama Desert, but radiation is still extreme, and water availability is restricted to fog and dew [54]. The biodiversity of the grit crust has not yet been fully understood, but important roles in CO_2_ fixation [53], bioweathering, initial pedogenesis [55], implications for astrobiology [56], and nature conservation [57], have been attributed mainly to lichens. Those lichens can best be described as crustose- to squamulose micro-lichens forming thalli not bigger than a few millimeters where often one specimen colonizes only a single grit, and they have a reduced set of morphological features (e.g., rare formation of apothecia), all of which exacerbates detailed studies. This is in contrast to any other known biocrust type or environment where lichens mostly form macroscopic, fruticose and/or large squamulose thalli in patches [58–60], while the micro-lichens of the grit crust form a coherent crust on the landscape scale [53]. In addition, insights into the ecology and phylogeny of lichen mycobionts and their photobionts from South America are rare [61, 62], but this holds particularly true for the Atacama Desert, which is considered a lichen hotspot, not only due to its fog oases [63–65] but also to its striking aridity gradient that correlates with latitude [66] and elevation [67]. Up to now, detailed insights into the microbial diversity of the grit crust are missing including information on symbiotic interactions such as between lichen mycobionts and their algal photobionts. It also remains an open question whether a single photobiont population or a diverse set of different photobionts or -lineages have been successful under the unique climate regime of the location leading to the broad, cohesive extent of the grit crust.

For this reason, we provide a polyphasic approach on grit crust lichens in order to answer questions about the organismic composition of the biocenosis, the lichen’s host-photobiont specificity, and the ecological role of their photobionts in the environment. We combine a Sanger-sequencing approach for taxonomic gene regions of myco- and photobionts based on low biomass picked directly from single lichens growing on single grits, which we micro-photographed, along with isolation of the photobionts. Those small-scale analyses were underpinned by metabarcoding and chlorophyll_a+b_ content determination alongside eleven locations of the grit crust community distributed over the 440 km^2^ spanning National Park Pan de Azucar. Due to the often described outstanding character of the grit crust and the great dominance of green algal lichens we combined classic biocrust research methods (metabarcoding, chlorophyll analysis) with analyses designed specifically for lichens and their trebouxioid photobionts (isolation, morphological studies of life cycles and chloroplasts, multigene-phylogenies). This unusual methodological combination helped us frame the grit crust's microbiome, to uncover the identity of the major lichen groups and their photobiont lineages, including the designation of unique potential photobiont species candidates. On the other hand, we intend to uncover the role of the grit crust’s lichen photobionts as *the* prime organism on the landscape scale amongst other biocrust-associated primary producers, which has so far never been exposed.

## Material and methods

### Sampling location

**Fig. 1:**
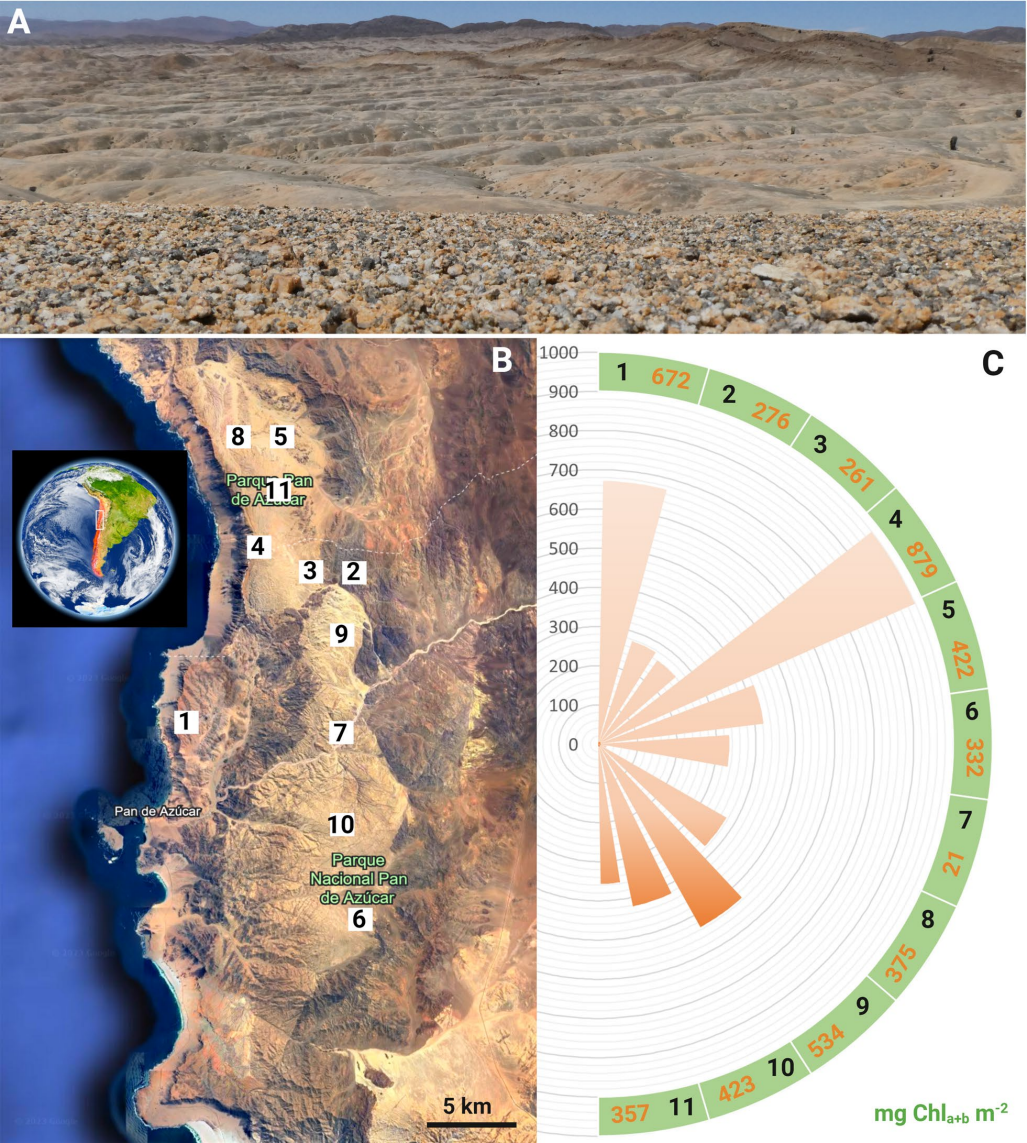
Grit crust landscape, sSampling locations and Chlorophyll_a+b_ m^−2^ content. **A** close-up photography of the grit crust in the foreground and the patchy distribution in the landscape in the background. **B** map of the National Park Pan de Azucar including the 11 sampling locations where plots were established for metabarcoding and chlorophyll determination. **C** Chlorophyll_a+b_ m^−2^ content of each plot

The National Park Pan de Azúcar, situated within the arid expanse of the Atacama Desert in northern Chile, spans approximately 440 km^2^ and encompasses a diverse range of ecological landscapes. The National Park Pan de Azúcar is a crucial site for scientific inquiry and preservation efforts in the context of arid and coastal ecosystems (Fig. 1A), which was highlighted during the research project EarthShape. As a result, geological and climatological information on the National Park can now be accessed [15, 25, 68].

### Sampling

In total, 11 homogeneously colonized plots with 1 m^2^ area each were established in the National Park Pan de Azucar (Fig. 1A, B) within comparable flat areas covered with densely colonized grit stones which appeared black [53, 55]. The corners of the plots were marked with long metal screws and the sides of the plots were arranged according to the cardinal points.

Within each of these plots the top centimeter of a 10 cm^2^ area was removed with a sterile brush and collected in plastic tubes for chlorophyll determination.

A second representative 10 cm^2^ area within the same plot was sampled as described above for a metabarcoding approach. Those tubes were filled with LifeGuard Soil Preservation Solution (Qiagen, Hilden, Germany) directly after sampling in order to preserve them.

Additionally, three samples of 50 g top-soil layer, each, were collected with a spatula directly beneath the 1 m^2^ plots and stored in plastic containers for the subsequent direct analyses of the lichens and their photobionts and the isolation of the latter.

The samples for chlorophyll determination and the taxonomic investigations of the lichens were stored air-dried at room temperature in the dark, while the preserved samples were stored at − 20 °C in the freezer until the beginning of the study.

### Chlorophyll extraction and quantification

In order to estimate the chlorophyll_a+b_ content of the grit crust, the top centimeter of a 10 cm^2^ area within the plots was removed with a brush and collected. For the chlorophyll extraction the dimethylsulfoxide (DMSO) method after Ronen and Galun [69] was applied which has been shown to be most suitable for biocrust samples [70].

### DNA extraction, amplification and sequencing of metabarcoding samples

Prior to DNA extraction, the LifeGuard solution was removed by centrifuging the samples at 2500 g for 5 min and discarding the supernatant. Due to colonization also of inner structures of the grit stones the samples were homogenized by beat-beating with steel beads (1 mm and 2.3 mm diameter) and a powder mill (Retsch, Haan, Germany) according to the DNeasy PowerSoil kit (Qiagen, Hilden, Germany), which was used for DNA extraction. The V4 region of the 18S rRNA gene (~ 390–430 bp) was amplified for eukaryotes using the universal eukaryotic V4 primers TAReuk454FWD1 and TAReukRev3 [71] and the ITS2 (~ 300–400 bp) region for fungi using the primer pair ITS3_KYO2/ITS4 [72]. The PCRs according to the conditions for each gene region, library preparation following the Tagsteady protocol [73] and sequencing was carried out by Fasteris (Plan-les-Ouates, Switzerland) using an Illumina MiSeq platform (2 × 300 bp). The full bioinformatic analyses were automatically processed using DeltaMP v0.6 [74] by high-performance computing (“Centre de Calcul de la Faculté des Sciences”, University of Neuchâtel, Switzerland). Raw Illumina reads were demultiplexed using cutadapt v4.1 [75] allowing up to 2 mismatches on the barcode sequences (8-nt long plus 2 to 4-nt long heterogeneity spacers at 5’-end). After stripping the primer sequences at the 5′-end with cutadapt (up to 3 mismatches allowed for 18S rRNA datasets and 5 for ITS dataset), only the amplified biological sequences were further analyzed. These first step produced four sets of reads, that is paired libraries (R1 and R2) for each orientation (i.e. R1 library starting either with the forward or the reverse primer). End of reads were stripped due to low quality. An automatic algorithm optimized this process in order to keep 75% of raw demultiplexed reads while maintaining an expected error on the remaining stripped reads below two and enough length to allow for pair end assembly. In the 18S rRNA dataset, forward orientation libraries were trimmed at 265 (R1) and 190 nt (R2) and reverse orientation libraries were trimmed at 270 (R1) and 225 nt (R2). In the ITS dataset, forward orientation libraries were trimmed at 260 (R1) and 170 nt (R2) and reverse orientation libraries were trimmed at 235 (R1) and 180 nt (R2). Amplicon sequence variants (ASV) were called using the *DADA2 R* package v1.26 [76]. Error models were built separately for each library and orientation, with turning the pool option on. Paired reads in R1 and R2 libraries were assembled with the *dada2 mergePairs* function, with a minimum overlap length of 10 nucleotides and a maximum of 1 mismatch in the overlap region. ASV originally with the reverse primer in the R1 library were reverse complemented using the *seqinr* package v4.2-16 [77]. The presence of chimera was double-checked first with the *dada2* function removeBimeraDenovo and with the UCHIME *de-novo* algorithm as implemented in VSEARCH v2.22.1 [78, 79]. The databases used to identify ASVs were PR2 v5.0.0 [80] for the 18S dataset and UNITE v9.0 (UNITE [81, 82]) for the ITS dataset. The UNITE database was augmented with sequences of isolates produced in this study (53 Lecanoromycetes and 49 Chlorophyta, see below). The taxonomy was assigned to each ASV using the VSEARCH option–usearch_global. When multiple best matches were found in the database, a consensus taxonomy was resolved at a threshold of 60%. The final ASV matrices contain 2.308.569 18S reads and 2.493.453 ITS reads distributed over 4.401 18S ASVs and 18.421 ITS ASVs. Statistical analyses were performed using R (v4.2.3) [83] and the package vegan (v2.5-7) [84], if not specified otherwise. For the 18S dataset ASVs not belonging to protist (Metazoa, Fungi and Embryophyta) were removed, as well as the ones having a percentage of identity below 70% with the database reference sequences and those that have less than 3 reads and that are present in only in one sample. For the ITS dataset, ASVs with a percentage of identity below 60% and that have less than 3 reads and that are present in only one sample were removed in addition to ITS sequences assigned to Chlorophyta in order to focus on fungi. General ecological functions of fungi were assigned based on the most updated literature for the corresponding ITS-derived ASVs consolidating the FungalTraits database [85]. Metabarcoding data were submitted to the European Nucleotide Archive (ENA) under the project code PRJEB72845 https://www.ebi.ac.uk/ena/browser/view/PRJEB72845.

### Isolation and cultivation of Lichen photobionts

Single grit stones with lichens growing attached to them were inspected under a binocular stereoscope (Stemi 508, Carl Zeiss Microscopy GmbH, Jena, Germany) under sterile conditions while small parts of the lichens were taken with a sterile metal needle and transferred into sterile PCR tubes filled with 200 µl Bold’s Basal Medium (BBM, [86]). The inoculated PCR tubes were subsequently streaked out on 12-well plates filled with solidified BBM (0.9%), sealed with parafilm and incubated in a culture room for four weeks at 17 °C, 30 µmol photons m^−2^ s^−1^ and a light:dark cycle of 18:6 h. Afterwards the growth of the colonies was inspected under the binocular stereoscope and transferred to fresh BBM plates until unialgal cultures were obtained. As a final step the 23 unialgal cultures were transferred to culture flasks filled with liquid BBM.

### Photo documentation and sequencing from samples and isolates

A direct PCR approach was applied to the micro-lichens growing on the grit stones forming the grit crust of the Atacama Desert. Single stones with lichens were selected under a binocular stereoscope, hydrated with sterile ddH_2_O and photographed using a 3D 4 K stereo microscope (VHX-7000, Keyence Deutschland GmbH, Neu-Isenburg, Germany). Subsequently a sterile needle was taken to pick small proportions of the lichens which were transferred to PCR tubes filled with 20 µl lysis buffer of the Platinum Direct PCR Universal Master Mix-Kit (Thermo Fisher Scientific Inc). The combination of photographing and extraction of biomass for molecular work allowed us to correctly assign DNA sequences of mycobionts and their photobionts to the morphology of the lichens. The DNA extraction of the biomass was carried out as described by the manufacturer including the setup of the mastermix and slightly modified [87]. For amplification of the ITS1 gene of the mycobiont the primers ITS1f [88] and LR3 [89] were used, for the green algal photobionts the nuclear internal transcribed spacer of the ribosomal DNA (ITS, ITS1, 5.8S, ITS2 was amplified using the primers ITS1T and ITS4T [90] with the identical lysate as DNA template.

In order to increase phylogenetic resolution for the isolated *Trebouxia* strains, *rbcL* was amplified using the primers *rbcL*1T and *rbcL*2T [91].

Success of amplification was checked by gel electrophoresis. PCR products that contained sufficient DNA concentration were cleaned using the NucleoSpin® Gel and PCR Clean-up Kit (Marchery Nagel, New England, Canada) and sent to Genewiz (Göttingen, Germany) for Sanger sequencing.

### Phylogeny of mycobionts and photobionts

The generated sequences were merged and the primers were trimmed using the software Geneious Prime (2022.01.1). Sequences for individual loci were assigned based on the NCBI GenBank dataset using the Basic Local Alignment Search Tool (BLAST) of Mega 11 [92]. Then, multiple sequence alignments were performed using the multiple sequence alignment method (Muscle) algorithm (Edgar 2004) implemented in Mega 11. The final datasets of the alignments consisted of 143 sequences of 983 bp comprising phylogeny of the ITS1 gene region of the mycobionts and a 2241 bp sequences comprising the concatenated ITS1, 5.8S, ITS2 and *rbcL* phylogeny of the photobionts derived from direct PCR on lichen material and the 23 isolates. For this dataset sequences from the study of Kosecka et al. [61] were also incorporated (Suppl. Tab1), since here sequences covering the same gene regions of photobionts of lichens from South America (Bolivia) were generated. We also incorporated sequences generated by Muggia et al. [50]. For each dataset the evolutionary model that was best suited to the database used was selected on the basis of the lowest Akaike Information Criterion (AIC) value and calculated in Mega 11 which was GTR + G + I for all. The phylogenetic reconstructions were calculated using NGPhylogeny.fr [93] with 500 bootstrap replications.

Besides Maximum Likelihood analyses (ML) also Bayesian Inference (BI) was calculated for each of these trees using Mr. Bayes 3.2.1 for the later analysis [94] as described in Kosecka et al. [61]. The tree topologies obtained by the BI method did not contradict the ML trees, thus only the ML trees are shown. Branches with bootstrap support and posterior probabilities > 80% were considered to be strongly supported and are marked by an asterisk.

Whenever possible, we used the numbering system for the designated photobiont clades according to Muggia et al. [50] and Kosecka et al. [61], but for clades which were newly designated during this study we used our own system. Phylogenetic trees were finally depicted in iTOL [95] and the resulting figures were edited in BioRender.

We also chose to prepare a phylogenetic reconstruction of the metabarcoding-generated ITS sequences on the ASV level which were assigned to *Trebouxia* instead of using the 18S ASV sequences because microalgae can show ecophysiological differences even when they are phylogenetically close, which is best displayed in ITS sequences for *Trebouxia* [50, 96]. Those sequences were combined with the full ITS-covering dataset from Muggia et al. [50] and the sequences generated during this study. The alignments were calculated using Mafft [93], manually trimmed in Mega 11 leading to a final dataset of 7.470 sequences of 561 bp length covering the ITS2 gene region. The phylogenetic tree was constructed using the GTR + G substitution model set to 500 replications at NGPhylogeny.fr [93].

### Microscopy

The 3D 4 K stereo microscope (VHX-7000, Keyencnce, Neu-Isenburg, Germany) was used as described above to capture in situ images during the growth of the isolated *Trebouxia* photobionts after 6 weeks on BBM agar plates.

In addition, light- and DIC microscopy were applied to take detailed images of the isolated *Trebouxia* photobionts using a BX51 (Olympus, Tokyo) coupled with a camera (MicroLive, Bremen, Germany) and MicroLive 5 software (MicroLive, Bremen, Germany).

Documentation of the developmental stages of the photobionts within the lichens were also made. This was carried out by removing a hydrated lichen thallus from the grit stones with tweezers under a binocular stereoscope and transferring it to a drop of water on a glass object slide. The thallus was afterwards slightly squeezed by pressing a cover slide on the object which was then investigated under the microscope.

To depict the chloroplast structure of the isolated *Trebouxia* confocal laser scanning microscopy (CLSM) was carried out using a Leica SP8 with an HC PL APO × 63/1.40 oil CS2 objective. Cells were immobilized on 1% (w/v) agar pads and excited at 458 nm. Fluorescence emission was recorded from 650 to 750 nm. Images were analyzed with Leica Application Suite X (version 3.5.2) and FIJI [97].

## Results

### Chlorophyll_a+b_ content as a proxy for photosynthetic biomass

On average 420 mg Chl_a+b_ per m^2^ were detected with a variability of 20%. In general, plots closer to the coast had higher Chl_a+b_ contents (e.g.: 1 = 672 mg m^−2^, 4 = 879 mg m^−2^) than others in distance to the coast (e.g.: 2 = 276 mg m^−2^, 3 = 261 mg m^−2^, 7 = 21 mg m^−2^) (Fig. 1C).

### Metabarcoding of eukaryotes and fungi associated with the grit crust

**Fig. 2:**
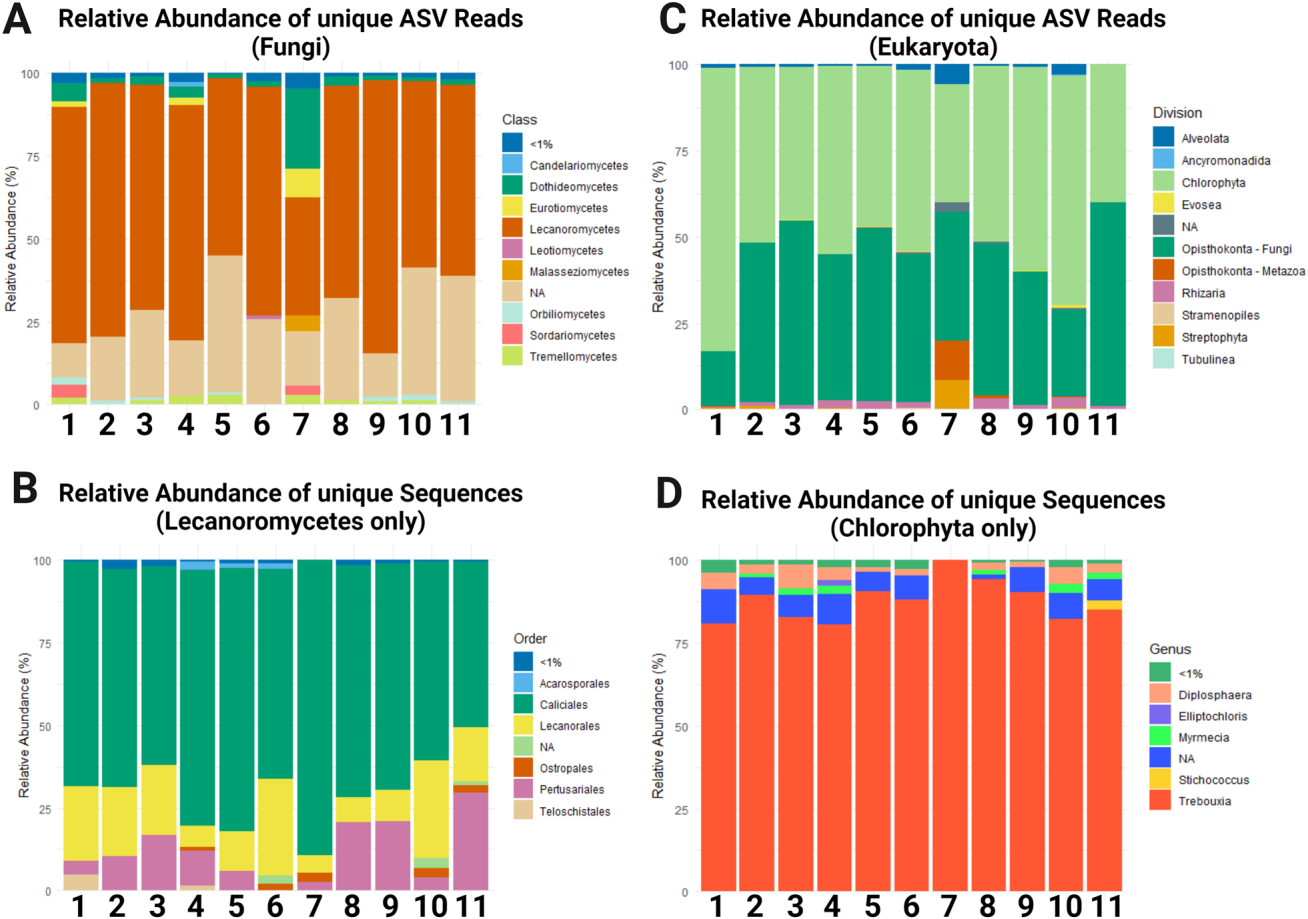
Metabarcoding. **A** Relative abundance of fungi-assigned ITS covering ASVs. **B** Relative abundance of Lecanoromycetes-assigned ITS covering ASVs. **C** Relative abundance of eukaryote-assigned 18S covering ASVs. **D** Relative abundance of Chlorophyta-assigned 18S covering ASVs

The eukaryotic community of the grit crust microbiome was dominated by Chlorophyta (18S, 50%) and Fungi (ITS, 20–60%) (Fig. 2), whereby the latter contained mainly lichenized fungi referring to lichens as the dominant organismic group. Among Chlorophyta, more than 80% of all 18S-derived ASVs could be assigned solely to the genus *Trebouxia* but other genera such as *Stichococcus*, *Myrmecia*, *Elliptochloris* and *Diplosphaera* were detected (Fig. 2).

More than 50% of all ITS-derived ASVs of the fungal microbiome were assigned to Lecanoromycetes (lichenized fungi), followed by less than 5% of each, Tremellomycetes (lichenicolous, parasitic fungi), Dothideomycetes (lichenized endophytes) and Eurotiomycetes (lichenized saprobiontic) (Fig. 2). Among the Lecanoromycetes the predominant order was Caliciales (60%) but also members of other lichens were detected such as Acarosporales, Pertusariales, Lecanorales and Teloschistales (Fig. 2).

Both targeted gene regions, ITS and 18S rRNA, covered algae and fungi but it turned out that, for example, Lecanoromycetes were underrepresented in the 18S rRNA dataset (61 ASVs) while the diversity of eukaryotic algae in general was underrepresented in the ITS dataset. For these reasons characterization of eukaryotic algae of the grit crust was best represented by the 18S dataset while fungal communities show a more realistic representation based on the ITS dataset, which has been commonly accepted [98].

### Phylogeny of ASVs assigned to Trebouxia

**Fig. 3:**
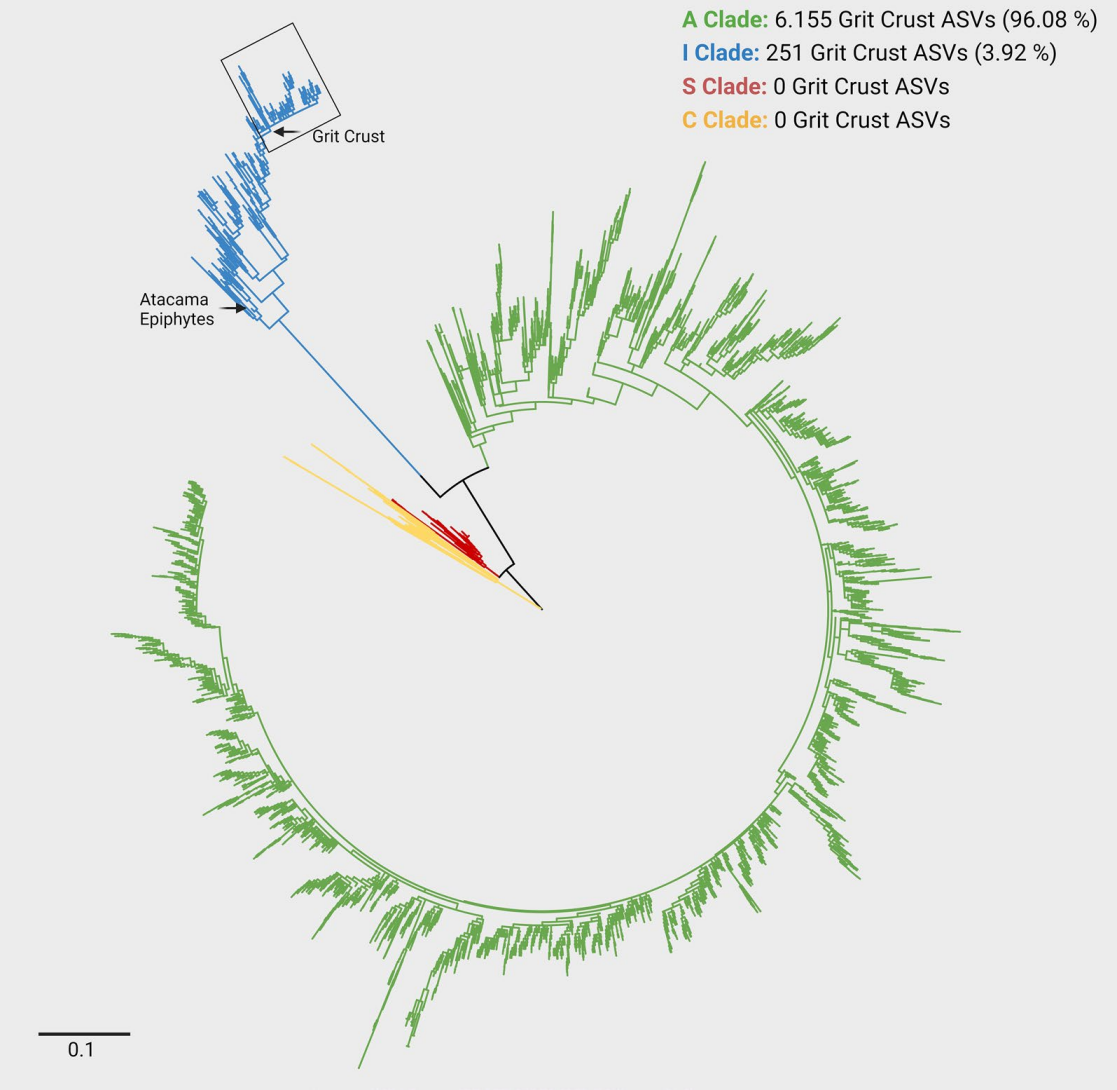
Phylogeny of the 6.406 *Trebouxia*-assigned ITS metabarcoding ASVs. The phylogenetic reconstruction represents the ITS2 covering sequences derived from metabarcoding of the grit crust and also includes the dataset of Muggia et al. [50], as well as the sequences created during this study based on the direct approach

The metabarcoding of the ITS2 gene region resulted in 6.406 ASVs assigned to *Trebouxia*. 96.08% of all grit crust *Trebouxia* ASVs could be assigned to the A clade, while 3.92% fell into the I clade. No ASVs of the C and S clade were detected. Within the I clade, the majority of ASV derived from the grit crust formed a separate cluster (Fig. 3, box and arrow) in distance to other sequences from epiphytic lichens (Fig. 3, arrow) sharing the same ecosystem. The grit crust derived ASVs of the A clade were distributed across the whole clade.

### Phylogenies of mycobionts, their uncultured photobionts and photobiont isolates

**Fig. 4:**
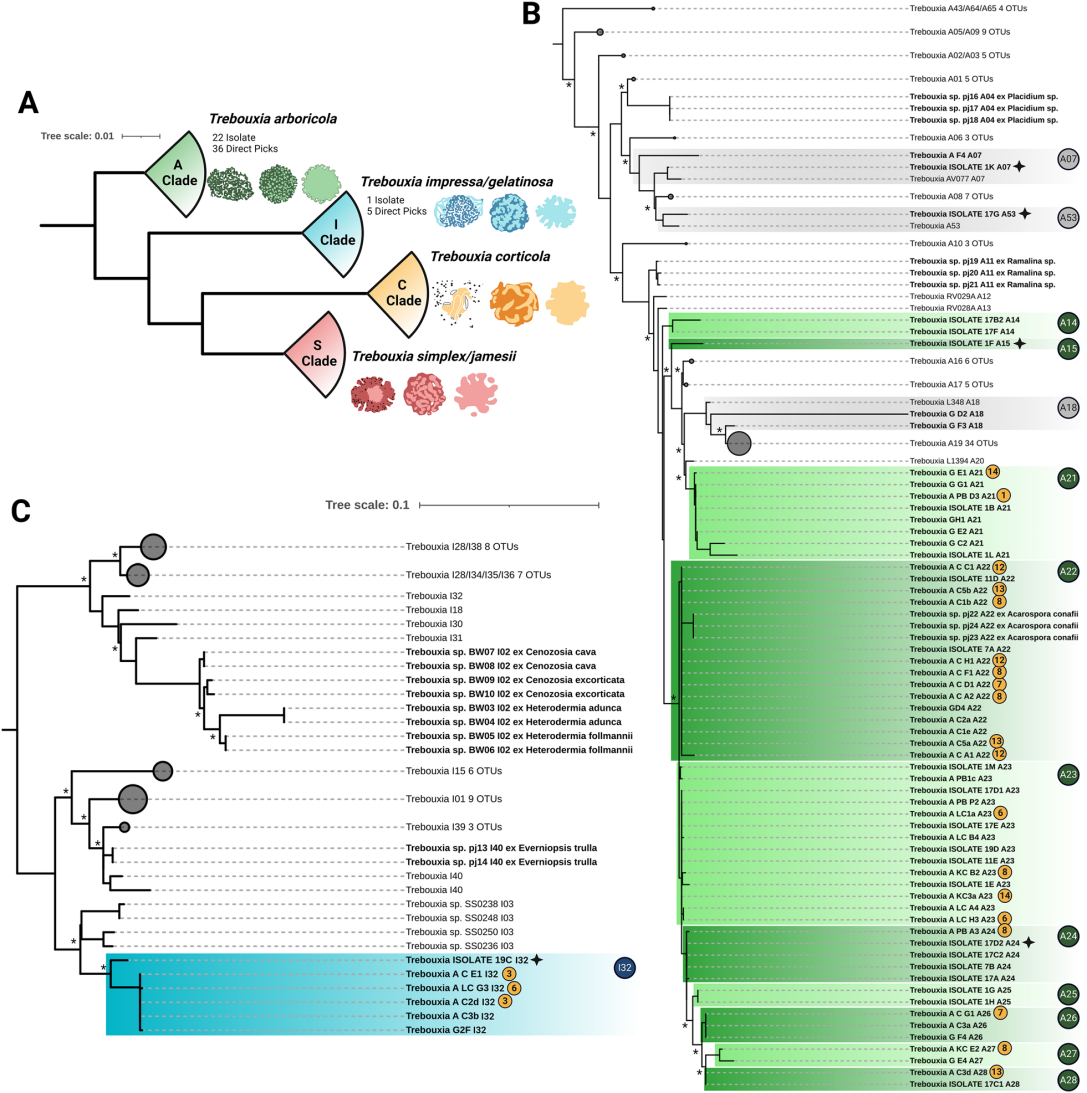
Concatenated *Trebouxia* phylogeny covering the ITS1, 5.8S, ITS2 and *rbcL* gene regions of sequences derived from direct sequencing of lichen material and isolates. **A** four major *Trebouxia* clades A (green), I (blue), C (orange) and S (red) after [50] and their assigned predominant pyrenoid and chloroplast structure reproduced after Bordenave et al. [48]. **B**, **C** phylogenetic reconstruction of the I clade (blue) and the A clade (green), respectively. Novel lineages are highlighted in color while already detected lineages are marked in gray. Stars mark potential species candidates for each novel lineage based on its phylogenetic positioning and morphological traits. Bold sequences without color boxes are derived from *Trebouxia* photobionts of epiphytic lichens sharing the same habitat but are not part of the grit crust. Orange circles indicate the number of the genus the corresponding mycobiont can be assigned to referring to Figs. 5, 6. Branches supported > 80% by Bayesian Inference (BI) and Maximum Likelihood bootstrap (ML) are labeled by an asterisk

**Fig. 5:**
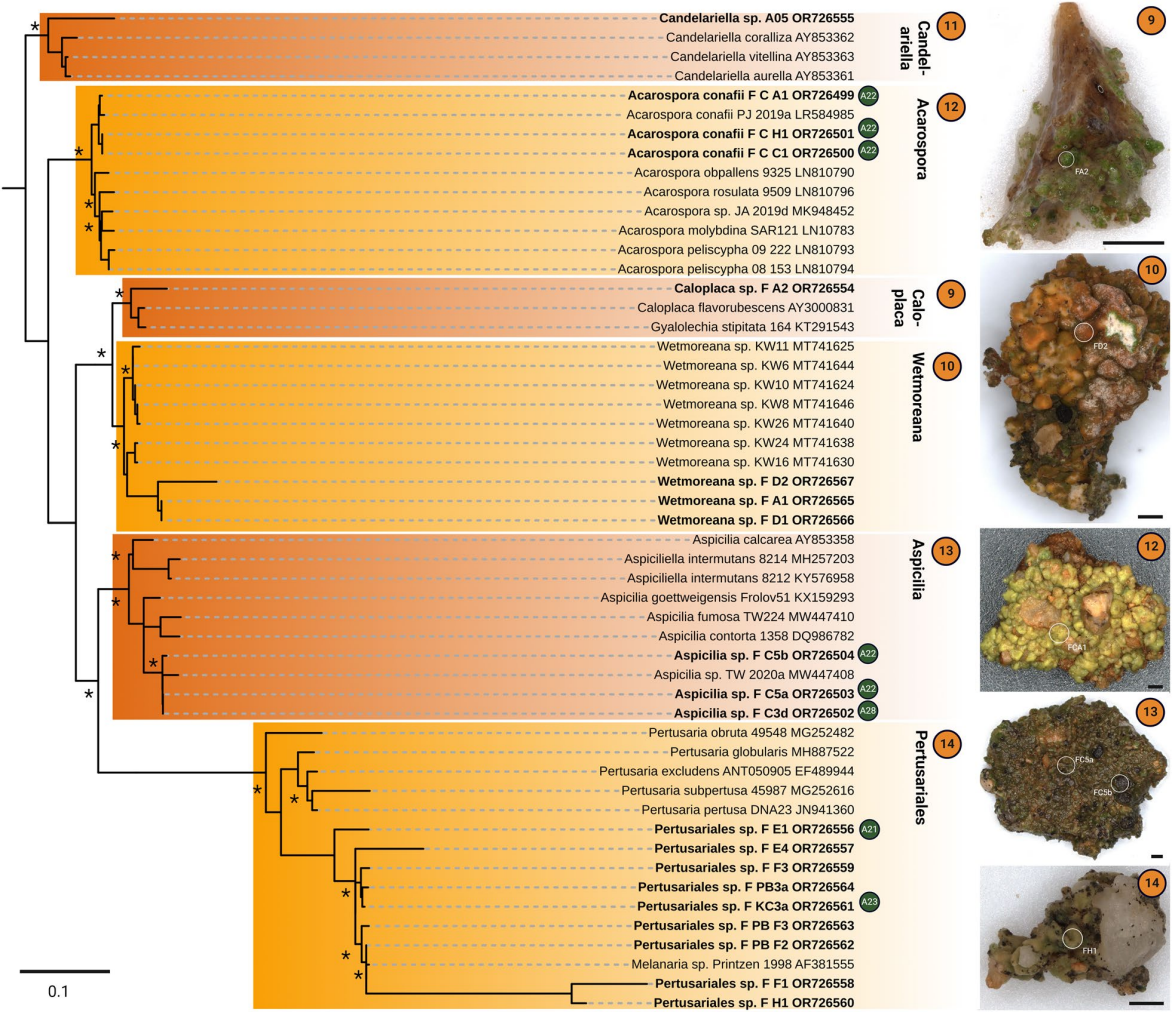
Grit Crust mycobiont phylogeny covering the ITS1 gene region derived from direct sequencing of lichen material. Orange circles number the mycobiont genera while green and blue circles indicate the lineage of the corresponding photobiont. Microphotographs show the exact origin of the biomass from which the mycobiont sequences and the corresponding photobiont sequences were generated. Scale bars are 500 µm. Branches supported > 80% by Bayesian Inference (BI) and Maximum Likelihood bootstrap (ML) are labeled by an asterisk

**Fig. 6:**
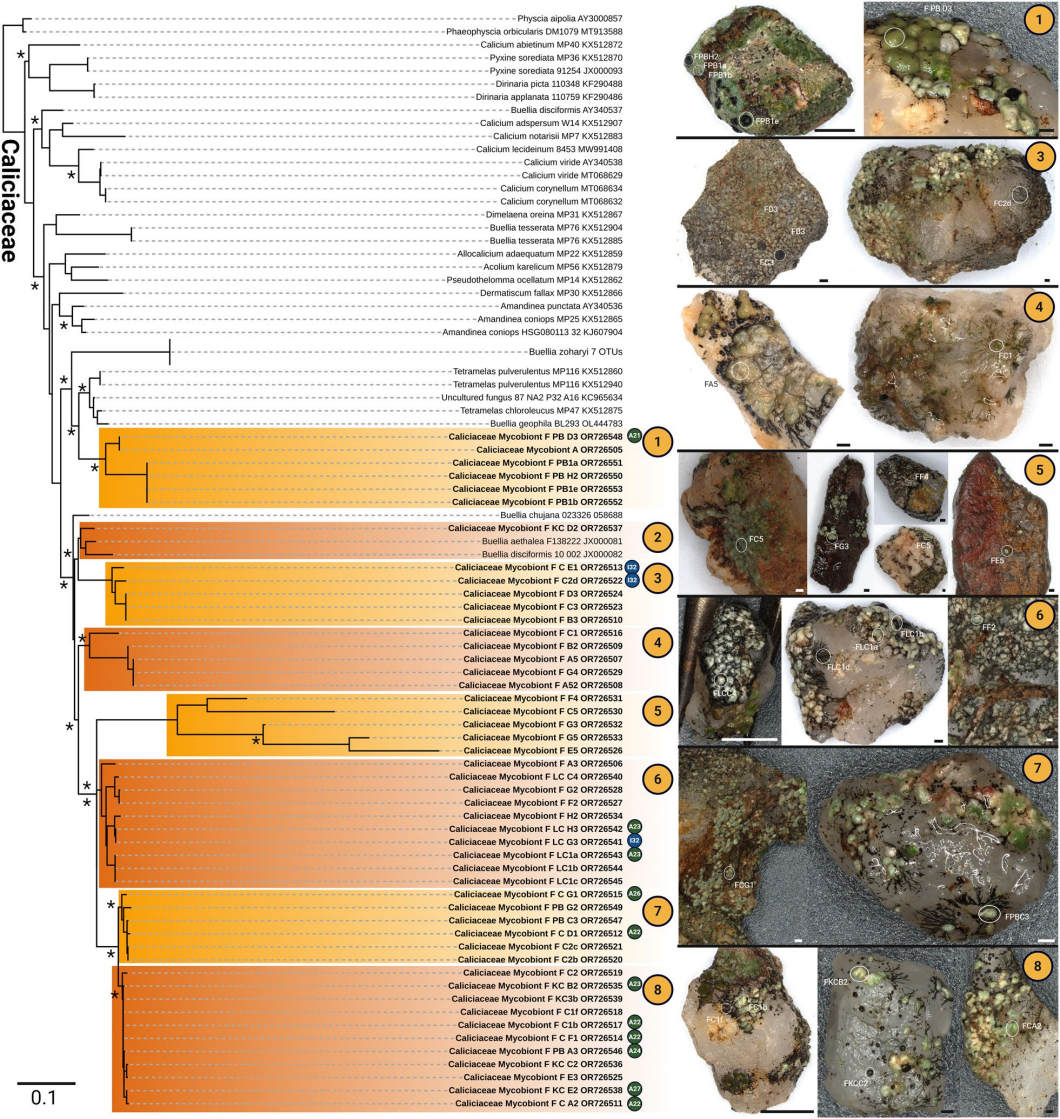
Grit Crust mycobiont phylogeny of Caliciaceae covering the ITS1 gene region derived from direct sequencing of lichen material. Orange circles number the mycobiont genera while green and blue circles indicate the lineage of the corresponding photobiont. Microphotographs show the exact origin of the biomass from which the mycobiont sequences and the corresponding photobiont sequences were generated. Scale bars are 1000 µm. Branches supported > 80% by Bayesian Inference (BI) and Maximum Likelihood bootstrap (ML) are labeled by an asterisk

The combination of microphotography of lichens on the grit stones and direct sequencing of the corresponding myco- and photobionts allowed for the determination of the phylogenetic position of 41 uncultured *Trebouxia*-photobionts and additional 23 isolates (Fig. 4) as well as 69 mycobiont sequences (Figs. 5, 6).

In detail, five uncultured *Trebouxia* photobionts and one isolate were assigned to the I clade (*T. impressa*/*gelatinosa*) based on the latest phylogenetic reconstruction of this genus [50] including the gene regions ITS1, 5.8S, ITS2 and *rbcL* (Fig. 4B). Together, those six sequences formed their own clade (I42) and other well-defined clades of photobiont sequences from the epiphytic lichens *Everniopsis trulla* (I40; [64]), *Cenocosia cava*, *C. excorticola*, *Heterodermia adunca* and *H. follmannii* (I02; [57, 65, 99]) sharing the same habitat were also detected within the I clade (Fig. 4B, bold).

Additionally, 36 uncultured *Trebouxia* photobionts and 22 isolates were assigned to the A clade (*T. arboricola*) (Fig. 4C). Within this A clade ten new clades were identified and a few sequences fell within three clades previously detected based on *Trebouxia*-sequences from Bolivia by Kosecka et al. [61] (A07, A53, A18). The ten new clades also comprised the photobionts isolated from *Acarospora conafii* (A22; [64]) but were not related to photobionts isolated from the terricolous lichen *Placidium* sp. (A04), the epiphytic *Ramalina thrausta* (A11) which both share the same habitat [64] or sequences generated from *Caloplaca* lichens from other areas of the Atacama Desert (A19; [63]).

Based on the corresponding lichen material used to generate the photobiont phylogenies 69 ITS1 sequences were generated (Figs. 5, 6), which were assigned to eight different clusters within the Caliciaceae representing potential new genera (Fig. 5) and to the genera *Caloplaca*, *Wetmoreana*, *Candelariella*, *Acarospora conafii*, *Aspicilia*, and one undetermined, potential new genus within the Pertusariales (Fig. 5). In some cases biomass and therewith DNA was extracted from black lichen prothallus, apothecia and lichen thalli of the same lichen in order to test if these structures belonged to the same species, which was always the case (e.g.: Fig. 5, FPBH2, FPB1a, FPB1b).

Whenever the sequencing of the myco- and the photobiont of the same lichen was successful, their affiliation was indicated in the corresponding phylogenetic tree in order to get preliminary insights into the photobiont-host specificity (Fig. 4B, C orange circles; Figs. 5, 6 green/blue circles). Based on these results a rather low photobiont-host specificity was detected, where lichens of several genera (*Acarospora conafii*, *Aspicilia*, Caliciaceae cluster 7 and 8) shared *Trebouxia* photobionts of a single clade (A22), but also that *Trebouxia*-photobionts of different clades (I32, A23) teamed up with lichen mycobionts of a single clade (Caliciaceae cluster 6).

### Trebouxia species candidates and developmental stages

**Fig. 7:**
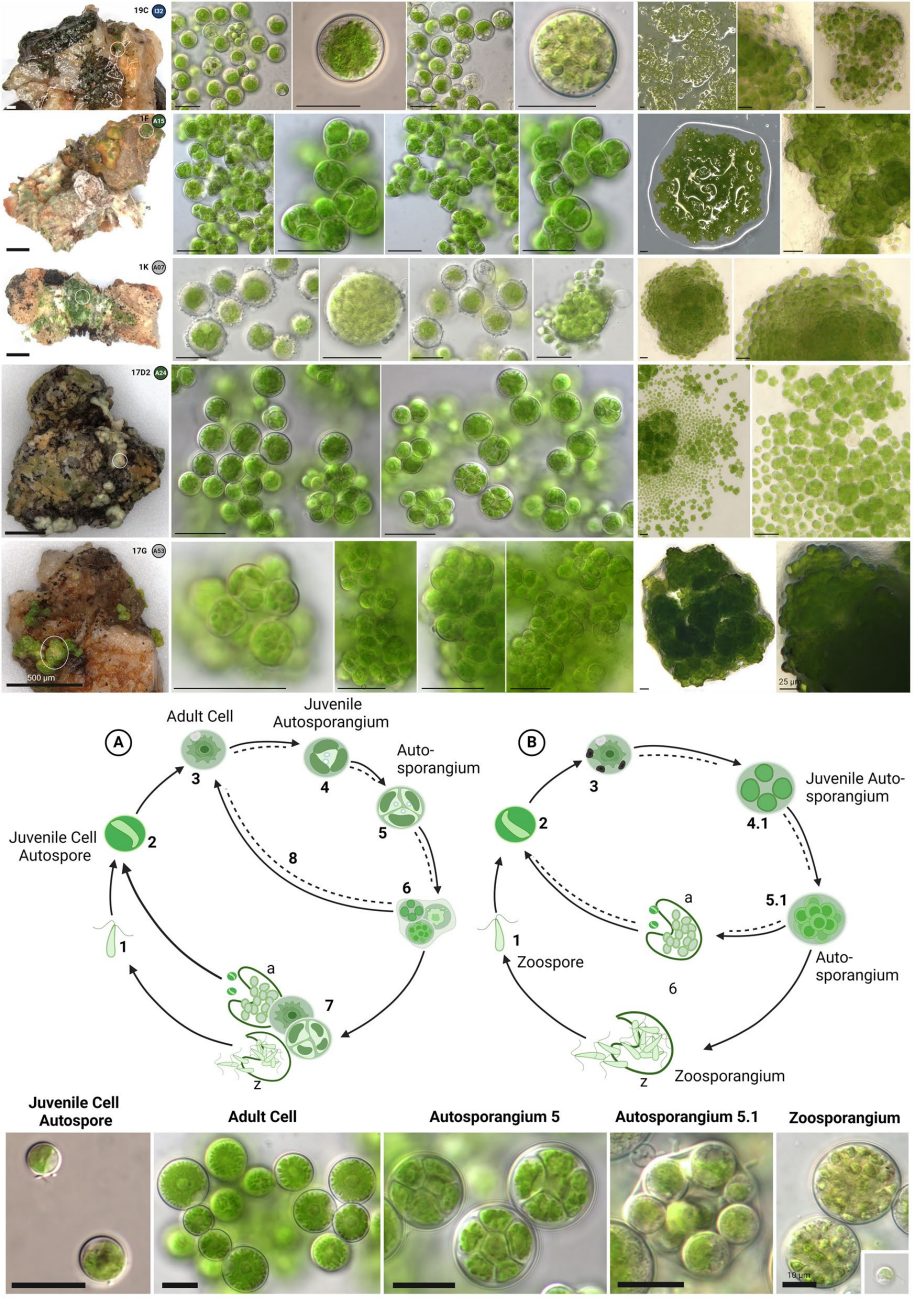
Potential *Trebouxia*-species candidates and their developmental cycles according to own observations after Friedl [44]. Top left shows the grit crust stones with the exact origin (encircled) of each isolate with the number of the isolate in the right corner as well as the *Trebouxia* cluster in the colored circle. Mid row shows microscopic images of each isolate. Right row shows 4 k microscopic images of the cultures grown on agar. Bottom: Broken lines: reproduction of *Trebouxia* within lichen thalli as interpreted from developmental stages seen in squashed preparations of algal layers of lichens. Cell cycle A: (1) zoospore; (2) juvenile vegetative cell (autospore); (3) adult vegetative cell; (4) protoplast division, chloroplast already divided during autosporangium (type 4) formation; (5) autosporangium (type 4) containing few autospores compressed within the sporangial wall; (6) autosporangium with autospores that begin to dissociate and where new protoplast divisions occur while the autospores that begin to dissociate and where new protoplast divisions occur while the autospores still adhere within the sporangial wall; (7) some autospores of stage 6 are developed into sporangia with numerous daughter cells which either escape as nonmotile autospores (a, autosporangium) or as free-swimming zoospores (z, zoosporangium) while other autospores are developed into an autosporangia (a) with few cells where new protoplast divisions occur; (8) autosporangium of 6 dissociates into single cells. Cell cycle B: (1) zoospore; (2) juvenile vegetative cell; (3) adult vegetative cell; (4) protoplast division, chloroplast already divided into four during autosporangium (type 4.1) formation; (5) cell containing numerous protoplasts (type 5.1 autosporangium); (6) development from stage 5.1 either into a sporangium with numerous nonmotile autospores (a) or into a zoosporangium (z). Microscopic images show all cell types / developmental stages

Potential species candidates within the designated lineages were evaluated based on morphological criteria of the isolates such as cell morphology, developmental stages in the lichen and in culture, chloroplast structure and their phylogenetic position deduced from the multigene phylogeny. Following this, five isolated species candidates could be identified: 19C (I clade), 1F, 1K, 17G and 17D2 (A clade) (Fig. 7).

**Fig. 8:**
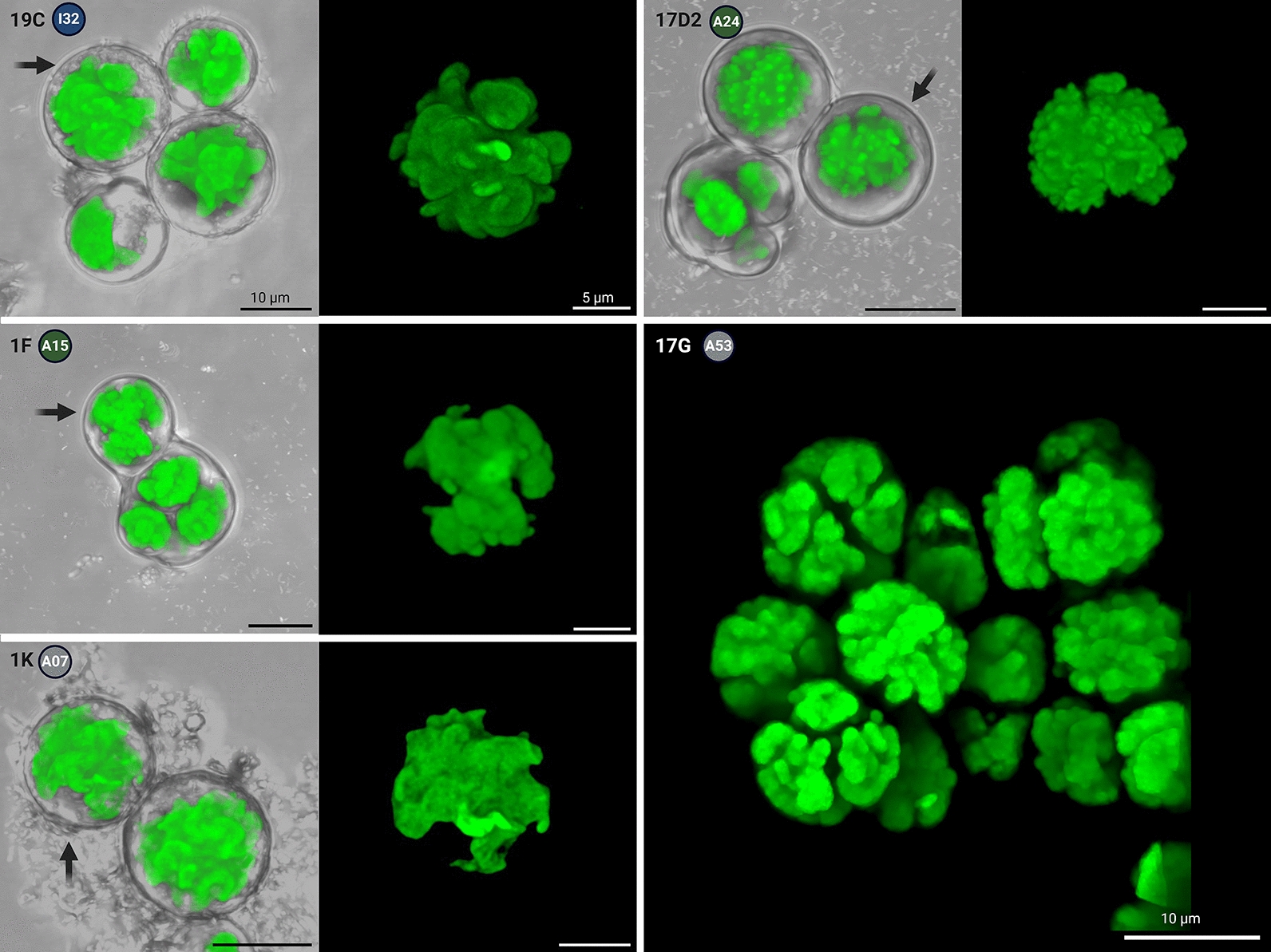
Confocal laser scanning microscopy (CLSM) showing the chloroplast structure of the *Trebouxia* sp. isolates. Gray images show the *Trebouxia* isolate and the assigned *Trebouxia* cluster in the colored circle. Arrows mark the cell which was visualized in detail

Isolate 19C shared a unique cluster (here named I32) with other sequences of uncultured *Trebouxia*-photobionts from the grit crust (Fig. 4B, C). The developmental stages of 19C correspond to cycle B according to developmental life cycles after Friedl [44] (Fig. 7), because irregular, spherical autosporangia only of the type 5.1 were formed in culture and in the lichen thallus. The cells were equally rounded, up to 23 µm in diameter, and showed a structured cell surface and a weakly lobed chloroplast filling up 2/3 of the cell, with distinct nucleus and often several, up to 10 µm large vacuoles (Fig. 7, top row). Zoosporangia reached up to 30 µm and were frequently detected. On agar plates a rather flat biofilm was produced with dispersed cells. The chloroplast was divided into almost regularly formed, rounded lobes (Fig. 8).

Isolate 1F had an isolated position (here named A15) within the multigene phylogeny (Fig. 4) and was further distinguished by highly aggregating cells of irregular shape, comparable to short pseudo-filaments resulting from incomplete deliberation of autosporangia of type 5 (Fig. 7), which was also observed in the lichen thallus. As a result 1F was assigned to the A life cycle (Fig. 7), but did not show zoosporangia and consequently also no zoospores in culture. The irregular, rounded cells had a diameter of 12 to 20 µm with a chloroplast which was fringed at the surface and filled the full cell (Fig. 7, second row). The isolate formed irregular colonies made of aggregated cells on agar which resulted in several layers and a conical structure. Confocal laser scanning microscopy revealed a lobed chloroplast with an irregularly formed fringed partitioning, sometimes forming small conical protrusions (Fig. 8).

Together with an uncultured *Trebouxia* from the grit crust the sequence of isolate 1K joined one other *Trebouxia* sequence (AV077; cluster A07) defined by Voytsekhovich and Beck [47] from *Lecanora albescens* from Ukraine. Cells of the isolate were round, up to 32 µm in diameter, with a broadly lobed, centered chloroplast filling up to 2/3 of the cell. An irregular structure on the cell surface was always visible. Developmental stages could be assigned to the B cycle with round autosporangia exclusively of the 5.1 type (Fig. 7) and up to 40 µm in diameter, which contained hundreds of round autospores (Fig. 7, third row). Zoosporangia and zoospores were not detected. Regular, conical to rounded colonies were formed on agar with loosely attached cells. The chloroplast was divided into irregular, thin lobes which extended into fringes near the cell periphery (Fig. 8).

The isolate 17D2 joined the cluster A24 which was only formed by *Trebouxia*-sequences generated from lichens of the grit crust (Fig. 4). The morphology of 17D2 was identical to all remaining isolates of uniquely defined clusters within the A lineage (A14, A1, A22, A23, A25, A28), thus this specific isolate was randomly chosen as a potential species candidate reflecting the morphology but not the genetic position of the named clusters/isolates. Cells of these isolates were rounded, only slightly aggregated, up to 22 µm in diameter with a centered chloroplast which filled most of the cell (Fig. 7, fourth row). Developmental stages followed cycle A (Fig. 7) with the formation of round, regular autosporangia of 25 µm diameter containing densely pressed autospores of angular shape. Even in young autosporangia the autospores mimicked the morphology of adult cells, including the shape of the chloroplast. Zoosporangia and biflagellate zoospores without stigma were rarely detected in culture. During growth on agar plates those isolates developed irregular colonies where the different developmental stages were separated, reflecting the age of the cells (Fig. 7, fourth row). The chloroplast was sometimes shifted and shallowly lobed. The chloroplast’s outer surface was made of small, regular protrusions resembling the ‘typical’ *T. arboricola* chloroplast morphology (Fig. 8).

Isolate 17G clustered together with a single sequence (A53) from a trebouxioid photobiont of *Myrolecis* spp. from Bolivia (Fig. 4) generated by Medeiros et al. [62]. The cells of the isolate grew densely aggregated made of cell packages. The cells themselves were irregularly ovoid to spherical with a diameter of 12 µm (Fig. 7, bottom row). Chloroplasts fully filled the cells and had a coarsely structured surface. The isolate followed life cycle A in cultivation and in the lichen thallus with autosporangia of type 5 (Fig. 7). The autosporangia were irregularly formed and comprised only a few cells with autospores that mostly stayed attached to the other autospores forming small cell packages. This was also reflected in the growth on agar plates where single colonies were formed which accumulated, resulting in round, dense structures reaching several mm. Zoosporangia and zoospores were not observed. Confocal laser scanning microscopy showed deep incisions of the chloroplast leading to irregular, thick lobes (Fig. 8).

## Discussion

Our holistic approach, applied to an unexplored area, enabled the characterization of biodiversity through different morphological and sequencing methods which, in combination, unveiled the hidden and impressive biodiversity of the photobionts as the integral part of the grit crust. This is in contrast to the majority of related studies on biocrusts, as they focus most often on the isolation or analysis of single phyla, thereby overlooking such vivid biodiversity. We demonstrated that an approach which spanned from microscopic observations on single micro-lichens to data generated from samples distributed over the 440 km^2^ wide National Park Pan de Azucar designated the symbiotic algal genus *Trebouxia* as the main primary producer of the grit crust, thriving under the unique conditions of the coastal Atacama Desert.

### The grit crust lichen community: saxicolous or biocrust associated?

Based on previous and ongoing work, it has been suspected that free living cyanobacteria and green algae only minorly contribute to the grit crust, while lichens dominate both in terms of biomass and function [53, 55]. It is not unusual for lichens to be a dominant component of biocrusts, some authors for example expect between 500 to 1000 species to be involved [100], albeit their biodiversity is usually low and restricted to a few species [101, 102]. Other studies using amplicon sequencing techniques on biocrusts only focused on certain phyla [7, 11, 103], did not detect interactions between Trebouxiophyceae and fungi [104], or only defined single lichen species as contributing locally [24]. For the grit crust this is a different situation compared to all other biocrust types known worldwide. This is due to the diverse and dominant group of lichens assigned to the family Caliciaceae with at least 8 genetically different clades as part of the community, complemented by genera such as *Candelariella*, *Acarospora*, *Wetmoreana*, *Caloplaca*, *Aspicilia* and *Pertusaria* (Fig. 5). This can be explained by the fact that the grits, being small rocks, favor a putative saxicolous lichen community over a soil crust-associated community. A detailed observation of these lichens shows that this is an oversimplification as all Acarosporales and a few Teloschistales ASVs can be assigned to *Acarospora conafii* and an undescribed species related to *Wetmoreana* genus. Both are strictly terricolous lichens from the same location growing only between the grits in the fine substrate and never on them [64, 99]. In addition, the core group of lichens contributing to the grit crust are lichens of the family Caliciaceae, which usually grow on diverse substrates such as bark, dead wood or rocks [105]. The rather graduated ecology of the non-epiphytic lichens from this habitat thus strengthens the definition of the grit crust as a transitional stage between a saxicolous community and a biological soil crust as previously outlined [3, 53]. Some members of the Caliciaceae, particularly those of the type genus, *Calicium*, are characterized by the presence of a mazaedium. This is an accumulation of loose, maturing spores covering the surface of the fruiting body, which is usually black, and either sits atop a long thin stalk, or rests on (or is sometimes immersed within) the surface of the lichen substrate. Consequently this results in a passive spore dispersal, a rare feature amongst Ascomycota [106]. However, many other Caliciaceae species generate spores in an apothecium, which typically resembles a flattened black disc (discoid, sessile and lecidiene ascomata) which is the case for all Caliciaceae-lichens of the grit crust (Fig. 4). In addition, most Caliciaceae-lichens of the grit crust have a well-developed prothallus made of a venous network of melanized, black hyphae stretching out several millimeters across the grit stones (Fig. 4; see also [53, 56]). In combination, the unique phylogenetic position of the Caliciaceae grit crust lichens based on the ITS gene region, the distinction of single, well supported clusters, the mutual absence of a mazaedium, their prothallus network and their extremotolerant ecology, will certainly justify the description of new genera and species. Most Caliciaceae species have a cosmopolitan distribution, although they are especially predominant in temperate and tropical montane areas, where they tend to avoid competition with other lichens and often grow in microhabitats uninhabited by other lichens [107]. This could explain the dominance of a unique Caliciaceae lichen subset thriving in such a habitat, which has been stable with an on-going aridity at least since the mid Miocene, 16–12 million years ago [108, 109], or even longer [110], leading to diversification over time.

Alongside the development of such an isolated lichen community are probably adaptations to the abiotic conditions such as the extreme radiation and the restricted but cyclic water input via probably fog and dew, which can be disadvantageous to other biocrusts [111, 112]. Also within the National Park Pan de Azucar differences in abiotic conditions affecting the grit crust microbiome exist, albeit mainly concerning water availability such as increased amount of fog water deposition close to the coast areas (climate data given for location 4 in [64] compared to topologically more enclosed inland areas (climate data given for location 7 in [68]). Since especially water availability is well known to shape lichen (-photobiont) communities this factor might explain diversity patterns but this is part of ongoing research in the area addressing for example fog water collection [113, 114].

However, adaptations of the organisms can on the one hand be supported, for example, by the melanized prothallus network of the grit crust lichens, which has been reported as a protection mechanism against UV radiation in other fungi and lichens from comparable ecosystems [115, 116]. On the other hand, ecophysiological adaptations of the grit crust community mirroring the abiotic conditions of the ecosystem have already been shown, identifying a comparably narrow ecological niche for optimum net primary productivity above 10 °C during the day, with no decrease in net photosynthesis up to at least 20 °C, 0.25 mm water content and a comparably high light compensation point (LCP) of 460 µmol m^−2^ s^−1^ [53]. Especially the exceptionally low water content required for optimum photosynthesis compared to other biocrust lichens is remarkable, since, for example, 6 mm were reported for *Acarospora schleicheri* from the fog zones of the Namib Desert [117] as well as 0.5–1 mm for lichen-dominated biocrusts from the Sonoran Desert [118]. However, the ecophysiological performance of lichens are most likely controlled, at least to a certain degree, by the lichen photobiont providing the carbon source for the whole lichen holobiont [119], supporting the theory that isolated lichen communities do not exist without an equally unique photobiont population [52, 61]. The low amount of water required for optimum photosynthesis of the grit crust might also be the key factor determining the dominance of green algae. In general, green algae and chlorolichens require minimal amounts of water for the activation of their photosynthesis and can do so even based on high relative air humidity alone, while cyanobacteria and cyanolichens depend on liquid water and higher amounts of water compared to green algae [120].

### *Trebouxia*: the dominant organism fueling the ecosystem of the grit crust

The lichen-forming algal genus *Trebouxia* is one of the best-studied symbiotic organisms associating with over 7.000 fungal species found in virtually all biomes linked to a diversification which took place during mid‐Paleozoic to mid‐Mesozoic [121, 122]. Getting closer to understanding this diversification process is a major challenge that was recently approached by defining four major *Trebouxia*-lineages A, C, I and S [48, 50]. According to this, certain lineages could be detected in a certain ecosystem, such as Icelandic cetrarioid lichens sharing photobionts of the S-clade [91], but a rather mixed subset was detected in most cases, especially when extreme ecosystems were considered [52, 61]. However, it has been proposed that the crown I-clade occupies warmer, as well as cooler and drier, habitats, i.e., Eastern Europe, while clade C occupies hot and humid climates in partially or exclusively forested habitats [123]. Moreover, the stem A-clade is dominant in only or partially forested habitats, but subsequently expanded in the Cenozoic Era to occupy regimes characterized by cooler and drier habitats—loosely coinciding with or after clade S invasion to similar climatic regimes [123]. Our study now sheds a new light on this concept since the Atacama Desert represents a climatically long-term stable, extreme, deforested, cool and dry environment from which hardly any lichen photobiont data exist. According to this, we found that the majority of grit crust associated lichens shared photobionts of the A-clade (96%, Fig. 3), while only some hosted an isolated subset of *Trebouxia* photobionts of the I-clade (4%; Fig. 3). This supports the findings of Nelson et al. [27], since on the one hand the cooler and drier habitat preference assigned to the I-clade is comparable to those found in the coastal zone of the Atacama Desert. On the other hand, the A-clade assigned *Trebouxia* members found in the grit crust might reflect the proposed photobiont subset after their expansion in the Cenozoic Era. However, Nelsen et al. [123] also argue that their concept does not give the full picture since some discrepancies occur (e.g. [124, 125]), which is probably due to an uneven dataset in public databases consolidated for the study as most recently outlined based on Bolivian lichens [61].

There, trebouxioid photobionts of all four clades including 16 new lineages among them were identified from 403 lichens of the Bolivian Andean vegetation providing the first study covering South American lichens and their photobionts according to the updated concepts. This data set also helped us to untangle the photobiont lineages of the National Park Pan de Azucar as great overlap could be found such as, for example, the assignment of photobionts of *Heterodermia* / *Polyplastidium* / *Leucodermia* and *Everniopsis trulla* from Bolivia and epiphytic relatives from the Atacama Desert sharing photobionts of the I-clade (Fig. 4; [61, 65]). However, those I-clade members from epiphytic lichens are only distantly related to those 4% of photobionts from the grit crust lichens and also formed a separated, unique lineages within all other I-clade photobionts (Fig. 3). The remaining 96% of the photobiont community of the grit crust could be assigned to the A-clade including photobionts from *Acarospora conafii*, *Wetmoreana* sp. and *Buellia*-related Caliciaceae lichens, all of which have formerly been assigned to this lineage form the same habitat [64, 99], but also from other habitats [126, 127].

Within each of those major A and I clades we could detect several new clades based on a multi-gene phylogeny such as I32 or A24 (Fig. 4), which are both the two most abundant representatives for the grit crust including the potential species candidates based on the isolates 19C (I32) and 17D2 (A24). The combination of the traditional, but still reliable, morphological comparison between isolated *Trebouxia* strains (Fig. 4) with the *Trebouxia* sequences obtained from direct amplification from thallus and Sanger sequencing (Figs. 7, 8), allowed us to demonstrate the genetic- but also morphological plasticity of the heterogeneous genus *Trebouxia*, which is reflected for example, in those two isolates. All potential species candidates showed significant differences in their cell sizes, chloroplast architecture, the formation of autosporangia, growth on agar and developmental stages in culture and in the intact lichen thallus (Figs. 7, 8). All of these characteristics were also identical for additional isolates sharing the same phylogeny-based clade. We also found identical phenotypic characteristics of isolates but with well-supported diverging genotypes (1B (A21); 17D2 (A24); 17C1 (A28); Fig. 4), showcasing that relying on either molecular analyses or morphological observations alone can lead to misinterpretations, as already proposed [50]. However, the unique set of isolated *Trebouxia* strains generated during this study allows a future formal description of the potential species candidates based on larger multi-gene phylogenies, which include for example, the *cox2* locus [50].

### Implications for the ecosystem

Our metabarcoding analyses highlight the presence of other green algae such as *Myrmecia*, or *Diplosphaera* (Fig. 2), both of which can be photobionts of lichens and/or free-living [128, 129]. We can exclude them from being lichenized since they were never found during the direct sequencing of grit crust lichen material. In addition, the metabarcoding derived 18S / ITS data did not indicate the presence of any lichen-forming fungi associated with e.g. *Diplosphaera*, such as *Verrucaria* or *Dermatocarpon* (Eurotiomycetes). However, it can not be ruled out that they might be the photobiont partner of epiphytic lichens of the environment or of grit crust lichen taxa which occur only very rarely. In addition, the metabarcoding approach we have chosen here provides a compromise between a comparable environmental study using standard primers and a lichen myco-/photobiont specific analyses due to biases introduced by primer selectivity [130]. The dominance of the green algal photobionts amongst the grit crust microbiome in general was also reflected in the high chl_a+b_ contents exceeding 800 mg m^−2^ at some plots with frequent fog influence close to the coast (Fig. 1B) and an average of 420 mg chl_a+b_ m^−2^ +- 20%. These values are comparable to well developed mesic biocrusts, e.g. from Germany (600–800 mg chl_a+b_ m^−2^, [70]), but exceed those found in comparable ecosystems such as the Negev or Namib Desert (< 150 mg chl_a+b_ m^−2^, [5, 131]). In general, the chlorophyll content of biocrusts undergoes seasonal fluctuations [5], and even higher contents can be expected for the grit crust during the winter season, where the fog water input and condensation strongly increase compared to the summer season [54, 64], during which the sampling for this study took place.

Those high chlorophyll contents can also reflect the productivity of a biocrust-ecosystem e.g. in terms of metabolic activity, net primary production and other ecosystem services which designates the grit crust as an active and principal driver of the habitat, which is otherwise mostly devoid of vegetation. More specifically, we showed that considerably the lichenized *Trebouxia* spp. substantially account for this, because this organism is responsible for the presence of its mycobiont partner, and therewith various other microorganisms all of which are sustained by the carbon provided by photosynthesis of the symbiotic alga.

**Fig. 9:**
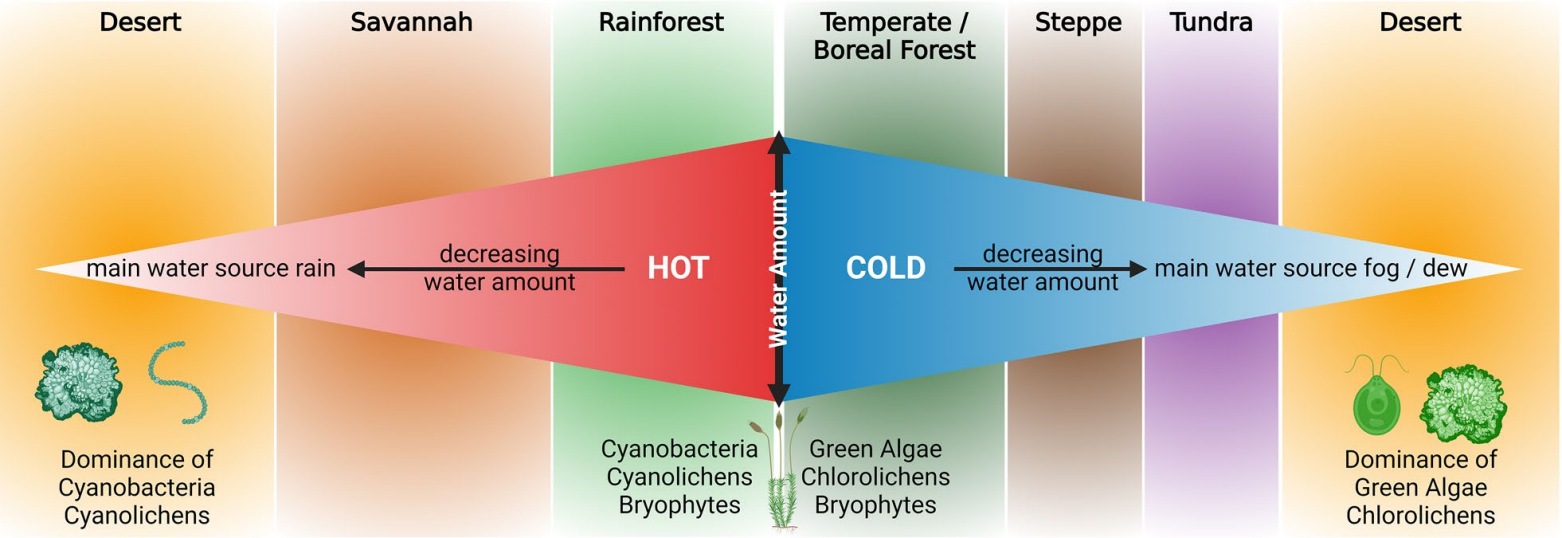
Graphical scheme of dominant biocrust-assigned cryptogams according to biomes. This scheme roughly illustrates which cryptogams are dominant in biocrusts according to temperature, water availability and -source. The grit crust of the Atacama Desert represents a biocrust type dominated by chlorolichens due to low water availability restricted to fog and / or dew as well as comparably low temperatures

In the context of biocrust research it has often been shown that water availability, water source and temperature as well as the resulting overall biome determine the most dominant organism(s) of the underlying biocrust [3], Fig. 9). Wherever water availability is not limiting, chlorolichens and mosses dominate, such as in temperate regions [132], but when cold temperatures dominate and desiccation occurs via frost, also cyanobacteria co-occur [9, 133]. However, wherever water availability is so limiting, that fog and dew become the major water sources, cyanobacteria disappear [134]. The grit crust as such extends the scale by defining cold deserts with low water availability and predominantly fog and/or dew as the main water source dominated by green algal lichens and green algae (Fig. 9). This theory is supported by ‘the enigmatic absence of cyanobacterial biocrusts’, which has recently been reported from the fog belt of the Namib Desert [134].

Our findings corroborate the globally unique character of the microbiome by designating the grit crust as the only known coherent soil layer with significant landscape covering impact, substantially ruled by diverse lineages of clade A of the symbiotic algal genus *Trebouxia* only.

## Outlook

For the first time, the symbiotic green alga *Trebouxia* has been shown to be the dominant primary producer of a vast area represented by the grit crust ecosystem in the coastal range of the Atacama Desert. This gives a new outlining to our ecological understanding of biocrusts which are dominated by lichens. These insights will allow future studies to target the resulting consequences for associated microorganisms such as a more detailed analyses of the lichen microbiome itself (e.g. amplicon sequencing of the lichen microbiome), metabolic exchanges from the trebouxioid photobiont to other microorganisms (e.g. metabolomics of the lichen microbiome) or direct interactions between the photobionts and the lithomatrix of the stones (e.g. bioweathering). Ecophysiological experiments with the isolated photobionts will now be possible in order to determine to which extent a certain *Trebouxia*-lineage or -species influences the overall carbon budget or the ecological niche of the grit crust lichens. In addition, the generated data can also help to detect and compare grit crust microbiomes outside of the National Park Pan de Azucar supported by approaches which aim to identify relations between certain mycobiont-/photobiont populations and spatio-temporal distribution patterns of those.

### Supplementary Information

Supplementary Material 1.

## Data Availability

All generated sequences were submitted to NCBI GenBank (https://www.ncbi.nlm.nih.gov/) and can be found under the accession numbers shown in Supl. Table 1. Metabarcoding data were submitted to the European Nucleotide Archive (ENA) under the project code PRJEB72845 https://www.ebi.ac.uk/ena/browser/view/PRJEB72845.
